# The prevalence of neovascularity in patients clinically diagnosed with rotator cuff tendinopathy

**DOI:** 10.1186/1471-2474-10-163

**Published:** 2009-12-21

**Authors:** Jeremy S Lewis, Syed A Raza, James Pilcher, Christine Heron, Jan D Poloniecki

**Affiliations:** 1 Therapy Department, Chelsea and Westminster Hospital NHS Foundation Trust, 369 Fulham Road, London, SW10 9NH, UK; 2 Therapy Department, St George's Hospital, London, UK; 3 St George's University of London/Kingston University, London, UK; 4 Radiology Department, St George's Hospital, London, UK; 5 Community Health Sciences, St George's University of London, London, UK

## Abstract

**Background:**

Shoulder dysfunction is common and pathology of the rotator cuff tendons and subacromial bursa are considered to be a major cause of pain and morbidity. Although many hypotheses exist there is no definitive understanding as to the origin of the pain arising from these structures. Research investigations from other tendons have placed intra-tendinous neovascularity as a potential mechanism of pain production. The prevalence of neovascularity in patients with a clinical diagnosis of rotator cuff tendinopathy is unknown. As such the primary aim of this pilot study was to investigate if neovascularity could be identified and to determine the prevalence of neovascularity in the rotator cuff tendons and subacromial bursa in subjects with unilateral shoulder pain clinically assessed to be rotator cuff tendinopathy. The secondary aims were to investigate the association between the presence of neovascularity and pain, duration of symptoms, and, neovascularity and shoulder function.

**Methods:**

Patients with a clinical diagnosis of unilateral rotator cuff tendinopathy referred for a routine diagnostic ultrasound (US) scan in a major London teaching hospital formed the study population. At referral patients were provided with an information document. On the day of the scan (on average, at least one week later) the patients agreeing to participate were taken through the consent process and underwent an additional clinical examination prior to undergoing a bilateral grey scale and colour Doppler US examination (symptomatic and asymptomatic shoulder) using a Philips HDI 5000 Sono CT US machine. The ultrasound scans were performed by one of two radiologists who recorded their findings and the final assessment was made by a third radiologist blinded both to the clinical examination and the ultrasound examination. The findings of the radiologists who performed the scans and the blinded radiologist were compared and any disagreements were resolved by consensus.

**Results:**

Twenty-six patients agreed to participate and formed the study population. Of these, 6 subjects were not included in the final assessment following the pre-scan clinical investigation. This is because one subject had complete cessation of symptoms between the time of the referral and entry into the trial. Another five had developed bilateral shoulder pain during the same period. The mean age of the 20 subjects forming the study population was 50.2 (range 32-69) years (SD = 10.9) and the mean duration of symptoms was 22.6 (range .75 to 132) months (SD = 40.1). Of the 20 subjects included in the formal analysis, 13 subjects (65%) demonstrated neovascularity in the symptomatic shoulder and 5 subjects (25%) demonstrated neovascularity in the asymptomatic shoulder. The subject withdrawn due to complete cessation of symptoms was not found to have neovascularity in either shoulder and of the 5 withdrawn due to bilateral symptoms; two subjects were found to have signs of bilateral neovascularity, one subject demonstrated neovascularity in one shoulder and two subjects in neither shoulder.

**Conclusions:**

This study demonstrated that neovascularity does occur in subjects with a clinical diagnosis of rotator cuff tendinopathy and to a lesser extent in asymptomatic shoulders. In addition, the findings of this investigation did not identify an association between the presence of neovascularity; and pain, duration of symptoms or shoulder function. Future research is required to determine the relevance of these findings.

## Background

Musculoskeletal disorders of the shoulder resulting in pain are very common, and after the 6^th ^decade shoulder pain appears to be more common than in any other region[1]. As the shoulder is used to position the hand to perform functional activities pain in this region is associated with substantial morbidity. Pathology of the rotator cuff muscle and tendon as well as pathology involving the subacromial bursa are considered to be principal causes of pain[2]. A review of anatomy and function of the rotator cuff and bursa and pathology involving these structures has recently been published[3]. Tendinopathy is a generic term to describe all forms of tendon pathology including structural changes such as; degeneration, tendinitis, partial and full thickness tears. It is associated with failed healing[4]. Tendinopathy may also be associated with biochemical changes[5,6]. In the shoulder rotator cuff tendinopathy may be associated with subacromial bursal changes[7].

The aetiology of rotator cuff tendinopathy is uncertain as is the cause of pain and proposed pathoaetiological factors have included; intrinsic tendon failure involving both tendon overload and underload, extrinsic irritation on the tendon, combinations of both intrinsic and extrinsic factors, postural variations, nutritional and systemic factors, evolutionary factors as well as genetics[4,8-14].

The classic pathoaetiological model of tendinopathy is inflammatory[15]. However this model has been challenged[16-20], and to a large extent the inflammatory model has been replaced by a degenerative model to describe the pathology in overuse tendinopathies, including those of the rotator cuff[21,22]. Alternative models have been proposed that have attempted to stage the pathology[4,8] as well as to explain the potential reasons for the pain[19,23,24]. However due to insufficient subject numbers, poorly designed studies, biopsy and chemical analysis performed after interventions such as; rest, electrotherapy modalities, NSAIDs, corticosteroid injections and rehabilitation, a robust understanding of the true pathohistology associated with acute through chronic rotator cuff tendinopathy specifically and other tendinopathies generally is profoundly limited, and it may be too early and presumptuous to abandon the inflammatory model until definitive data are presented. In support of this, the signs of cells classically associated with inflammation have been reported in small but not large rotator cuff tears[25] and in the bursal tissue of patients with constant and night pain, but not in patients with pain experienced only on movement[26]. It is highly likely that the pathoaetiology and pathohistology of rotator cuff tendon pain and dysfunction are complex and multifactorial, as well as being time and presentation dependent.

A non inflammatory chemical irritation of the neural tissue associated with tendons has been proposed as a potential mechanism of pain production [5,17,18,24,27,28]. The potential for chemical irritation in rotator cuff tendinopathy is supported by studies that have demonstrated sensory innervation and the presence of nociceptive free nerve endings (Aδ and C)  in the subacromial bursa[29-31]. In addition, high concentrations of chemicals that may stimulate these nociceptors have been identified in patients with clinical signs of rotator cuff tendinopathy and subacromial pain syndrome[5,32]. These include; substance P and calcitonin gene-related peptide[29], the matrix metalloproteinases; MMP1 and MMP9, the cyclooxygenase (COX) enzymes; Cox-1 and Cox-2, tenacin-C, and the cytokines; IL-1β, IL-6, TNFα and vascular endothelial growth factor (VEGF) in the bursal and tendon tissues[5,28,33-36]. A positive correlation between the concentration of some of these chemicals and the intensity of pain has been reported[32]. Findings from animal studies have demonstrated that the combination of substance P and interleukin-1 leads to the development of intense neovascularisation[37]. Neovascularity associated with tendons is characterised by the formation of microvascular networks in and around tendon tissue[38,39], and may be associated with neural tissue that may possibly be associated with tendon related pain[40]. Colour Doppler ultrasound[38,40,41] and power Doppler ultrasound[39] have been used extensively to identify neovascularity associated with tendon pathology and have become an established method based on the principal that blood flow rates in normal tendon are relatively low[42]. Power Doppler ultrasound is a well-recognised and long-established technique for detection of soft tissue hyperaemia or neovascularisation. Newman et al demonstrated that power Doppler consistently determines hyperperfusion associated with musculoskeletal inflammatory disorders[43]. This depiction of increased vascularity, ranges from frank tissue blush to isolated new peritendinous and peribursal vessels. The tissue blush is thought to represent very small vessels (microvascular flow)[43].

In the past decade a relationship between neovascularity and tendon pain has been proposed [38,44-46]. Alfredson et al[40] used a combination of clinical assessment and ultrasonography to compare 25 tendons in 24 people with painful midsection Achilles tendinopathy, with 20 tendons from 14 subjects without symptoms. Grey scale ultrasound and colour Doppler ultrasound were used to respectively investigate the tendon structure and blood flow within the tendon. Grey scale ultrasound in the subjects without symptoms revealed a normal tendon structure. Although the study lacked sufficient information on the source population the reported findings from the 25 symptomatic tendons involved a localised widening of the tendon, together with irregular fibre structure and focal hypoechoic areas corresponding with the painful mid-section of the tendon were identified. In all 25 painful tendons, neovascularisation was identified in the area of the grey scale tendon changes. Neovascularisation was not observed in the 20 pain-free tendons.

Ultrasound utilizes sound waves in the range of greater than 20,000 Hz normally inaudible to human ears. Ultrasound travels in longitudinal waves and when they strike human tissue produce echoes at tissue interfaces[47]. Although some of the waves get absorbed others are reflected back to skin where the US probe detects and processes the waves to construct an anatomic image. For visualisation of musculoskeletal structures, the most useful frequencies are between 7 and 12 MHz. Ultrasound utilizes the *Doppler Effect*, in which the reflected frequencies from an object appear to change as the object travels toward or away from a reference point. Using this principal, information regarding blood flow may be obtained[48]. Colour Doppler ultrasound depicts blood flow as a colour map superimposed on the gray-scale image in which the colours represent the direction and mean velocity of blood flow (i.e. towards or away from the transducer). Power Doppler displays the integral strength of the Doppler signal as a single colour and usually offers no information on direction. It tends to be more sensitive at demonstrating low volume/velocity flow, making it possible to visualise small vessels[49].

The identification of neovascularity in painful Achilles, patellar and lateral elbow tendons and the early but nonetheless encouraging results from treatments directed at the neovascularity [38,50,51] may offer a potential method of treating painful tendons once more robust research evidence is available. The prevalence of neovascularity in patients with a clinical diagnosis of rotator cuff tendinopathy is currently unknown. As such the principal aim of this study was to investigate the prevalence of neovascularity in the rotator cuff tendons in subjects with unilateral shoulder pain clinically assessed to be rotator cuff tendinopathy. Secondary aims included an investigation of the relationship between neovascularity and (i) pain, (ii) duration of symptoms, and (iii) shoulder function.

## Methods

### Study design

The study design was a case series designed to investigate the prevalence of neovascularity in the rotator cuff and subacromial bursa in people with a clinical diagnosis of unilateral rotator cuff tendinopathy.

### Ethics

Permission to conduct this study was granted by the Wandsworth Research Ethics Committee, St George's University of London, London, UK. Research Ethics Committee reference number: 07/Q0803/1. All subjects signed witnessed informed consent documents and were aware of all their rights, including the right to withdraw from the study at any stage of the investigation.

### Setting and data collection

The investigation took place in a dedicated diagnostic ultrasound clinic in a major acute NHS (National Health Service) teaching hospital in London, UK. Data collection took place from July 2007 to March 2009.

### Participants

Potential participants included people aged between 30 to 75 years with a diagnosis of unilateral shoulder pain who were referred to the radiological department by their physical therapist, orthopaedic surgeon or general medical practitioner with a special interest in musculoskeletal disorders for a routine diagnostic ultrasound scan of the rotator cuff.

### Inclusion/exclusion criteria

Inclusion criteria for the participants were; (i) unilateral shoulder pain, exacerbated during movement located in the C4/C5 dermatome, (ii) age range between 30 to 75 years, (iii) pain experienced on flexion and/or abduction, (iv) pain experienced during the Neer Sign and/or Hawkin's Test, (v) pain on resisted abduction and/or resisted external rotation and or during the 'full cans' test and/or the 'empty cans' test, and (vi) passive shoulder range to be greater than active range. The exclusion criteria were; (i) the reproduction of shoulder pain during physiological cervical spine movements, (ii) a history of shoulder subluxations and/or dislocations, (iii) an injection to the shoulder in the past 12 months, (iv) physiotherapy, osteopathic or chiropractic treatment to the shoulder within the past 12 months, (v) recent or previous shoulder girdle fractures, (vi) open wounds or skin infections in the region of the shoulder, and (vii) concurrent involvement in another research investigation.

### Procedure

Potential participants who were being routinely referred for a diagnostic ultrasound scan of the rotator cuff were informed of the investigation and if interested in learning more were provided with a participant information document by their referring physical therapist, orthopaedic surgeon or general medical practitioner. In addition, potential participants were encouraged to discuss possible participation with family, friends, healthcare workers and if so wished with the chief investigator. The design ensured that those wishing and those not wishing to participate, and those fulfilling and not fulfilling the inclusion criteria would receive their scans at the same time point following referral. Those wishing to participate or discuss participation in more detail met with the chief investigator prior to the ultrasound scan. On the day of the scan, at approximately one hour before the ultrasound scan was performed, the participant met with the chief investigator to discuss the study and answer any further questions. Any potential participant not wanting to participate would proceed directly to have their scans. Those agreeing to participate signed witnessed informed consent documents. Following this, the chief investigator performed a routine clinical investigation involving; impingement tests, shoulder range of movement, shoulder resisted movement tests and cervical screening procedures, principally to ensure compliance with the inclusion and exclusion criteria on the day of the ultrasound scan. In addition to this each participant provided routine demographic information and completed the Oxford Shoulder Score[52].

### Clinical tests and functional measurements

The following are descriptions and interpretations of the clinical procedures used to include and exclude the potential participants.

#### Neer Sign

The patient is seated and the examiner standing. Scapular movement is restricted by one of the examiners hands firmly placed on the scapula. The examiners other hand forces the patient's arm into shoulder elevation (described as somewhere between flexion and abduction). A positive response was pain around the acromion during this manoeuvre[53].

#### Hawkin's test

Hawkins and Kennedy[54] described a test that involves flexion of the humerus to 90° and forcibly internally rotating the shoulder. A positive response was pain around the acromion during this manoeuvre.

#### Resisted abduction 30°

Liu et al [55] reported that the peak abduction moment for the supraspinatus tendon occurs at 30° of abduction. A positive clinical response to an isometric contraction at this point in the range was pain under or lateral to the acromion.

#### 'Full can' and 'empty can' test

Jobe and Jobe[56] described a supraspinatus test where the arm is placed at 90° of abduction in neutral rotation (full can) and resistance to elevation is applied. The shoulder is then internally rotated and angled forwards by 30° so that the thumb faces the floor (empty can) and resistance to elevation is repeated. Increased pain or increased pain and weakness during the 'empty can' test was positive.

#### Shoulder active and passive range of movement

Shoulder flexion (assessed in the sagittal plane performed with the thumb facing upwards) and abduction (assessed in the plane of the scapula with the thumb facing upwards) range were measured actively in both shoulders and passively (only in the symptomatic shoulder), in standing. Active movement was measured at the first point of pain and passive movement, was assessed at the end range of movement or at the point an increase of pain did not allow any further movement. Active range was measured using a gravity dependent inclinometer as has been described previously[57]. Passive range was not measured with the inclinometer but was recorded as equal, less than or more than the active range.

#### Cervical screening procedure

No clinical test has been documented that conclusively substantiates the relationship between cervical structures and shoulder symptoms. In an attempt to exclude cervical referred shoulder pain, each subject was requested to actively flex, extend and rotate the cervical spine to the left and right to the end of their available range to determine if these movements reproduced the individuals shoulder pain[58]. If symptoms were produced in the shoulder the participant would be excluded.

#### Oxford Shoulder Score

The Oxford Shoulder Score is a validated 12 question shoulder disability completed independently by the individual, with a score ranging from 0 to 48, where 0 represents maximal disability and 48 no disability[59].

### Ultrasound procedure

Ultrasound of both shoulders was performed using a Philips HDI 5000 sono CT (Philips Medical Systems, Surrey, UK) with the patient sitting upright and the examiner scanning from the front. A linear high frequency probe (5-12 MHz) was used with the musculoskeletal shoulder setting preset with the room darkened to enhance monitor visualization. The ultrasound scan was performed using a standard protocol beginning with examination of biceps tendon in transverse and longitudinal planes with the patient's forearm or hand resting in a supinated position on thigh. Following this the acromioclavicular joint was assessed in the same position. The subscapularis tendon was then examined with the patients arm externally rotated. Imaging of supraspinatus tendon was performed by asking the patient to place the hand in the back pocket area with the palm facing toward the gluteal muscles while keeping the elbow directed posteriorly. The supraspinatus tendon was examined in the transverse and longitudinal planes. Vascularity was then assessed in both planes with power Doppler optimized for low flow imaging and a pulse repetition frequency of 700 MHz. The posterior glenohumeral joint, infraspinatus and teres minor were examined with patient's arm across the chest with the hand placed on the opposite shoulder.

### Blinding of the interpretation of the ultrasound scans

To reduce knowledge of the side of symptoms as a source of bias, a third radiologist who was not present at the time of the ultrasound investigation, and was therefore blinded to the clinical symptoms and side of shoulder pain, assessed the images for the presence of grey scale changes and neovascularity. The third radiologist was provided with the hospital identification number of the participants and the ultrasound studies were then reviewed on hospitals digital imaging system without reference to any clinical details. The anonymization was performed by disabling any clinical information (i.e. request form or reports) available on the electronic patient archiving and communication system information window.

After the third radiologist completed the blinded assessment of the ultrasound scans his findings were compared with the initial reports. It was planned that any uncertainties would be resolved by consensus between the third radiologist and the two involved in the scanning. This arbitration process was not required. There was good consensus between the three radiologists in detecting and/or scoring neovascularisation. This has been reflected in other studies. Sengkerij et al demonstrated excellent inter-observer reliability when scoring neovascularisation in the mid portion of the Achilles tendon[60].

### Description of grey scale ultrasound and power Doppler neovascularity findings

Grey scale ultrasound appearances of tendinopathy include swelling of affected tendon and abnormal tendon echotexture with a heterogeneous hypoechoic appearance[7]. Tendon swelling is interpreted as focal or more often diffuse increase in tendon thickness. In the case of the supraspinatus, a difference in tendon thickness of 1.5 to 2.5 mm between the affected and unaffected side or an absolute thickness of more than 8 mm is thought to indicate tendinopathy[61]. The abnormal echotexture represent subtle fibrillar tears and area of mucoid degeneration interspersed with reparative process in tendon substance. A partial thickness tear is diagnosed on ultrasound when a true defect or cleft within the tendon substance is detected in both longitudinal and transverse planes. A full thickness tear extends from the bursal to articular surface and associated with other indirect signs[7]. We devised a simple grading system to describe the amount of neovascularity identified. Grade 0, no neovascularity; Grade I, mild neovascularity with no recognisable vessels; Grade II, identifiable vessels involving less than 50% of the area imaged; and, Grade III, identifiable prominent vessels involving more than 50% of the area imaged. In addition to the grade, the location of any observed neovascularity was also recorded. A positive correlation exists between tendinopathy and neovascularisation[44,62]. However, the exact significance of this correlation is yet to be determined.

### Statistical methods

Associations between neovascularity and (i) pain and (ii) the Oxford Shoulder Score were tested. Fisher's Exact test was used to test the association with dichotomous measures. For continuous measures Wilcoxon's test was used. All tests were two-tailed and statistical significance was set at p = 0.05.

### Sample size calculation

There was a conceptual assumption that the prevalence of neovascularity would be 0% in the shoulders without symptoms. This was based on previously published research[40]. We estimated a prevalence of 30% for the presence of neovascularity in people with symptomatic shoulders with a clinical diagnosis of rotator cuff tendinopathy. Based upon this hypothesis it was estimated that 20 subjects with unilateral shoulder symptoms would have a 58% power to show statistical significance.

## Results

**Figure 1:**
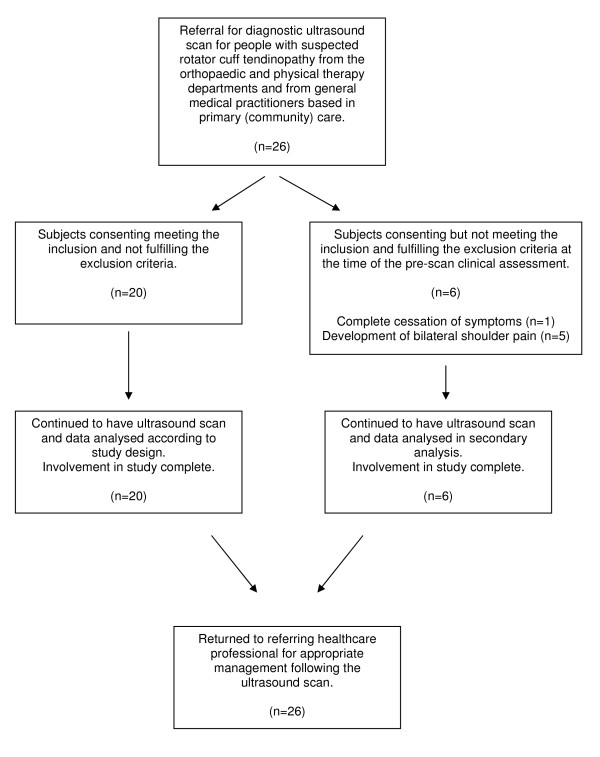
**Participant progression through the study**.

Twenty-six participants with unilateral shoulder pain who were consecutively referred for an ultrasound scan and agreed to participate in the investigation formed the study population. All referred potential participants were willing to participate in the investigation. The average wait between the referral to the ultrasound scan and the day of the scan was at least one week. During this time symptoms changed in six participants. This was detected during the pre-scan clinical evaluation. These changes ranged from complete cessation of symptoms (one subject) to development of bilateral pain (five subjects). These six individuals continued to have an ultrasound scan as requested by their referring healthcare professional. Although their ultrasound findings were recorded, the results of these six people were not included in the main data analysis as the design of this investigation was to use the asymptomatic arm as a control. Figure 1 details the participants' progression into and through the study.

The mean age of the 20 subjects forming the study population was 50.2 (range 32-69) years (SD = 10.9) and the mean duration of symptoms was 22.6 (range .75 to 132) months (SD = 40.1). Additional demographic and clinical data for the 20 subjects included in the analysis are detailed in Table 1.

**Table 1:** Demographic and clinical information for subjects included in analysis

Subject	Age (years)	Gender	Painful side	Dominant side	Duration of symptoms (months)	Pain at rest	OSS 0=worse 48=best	Grey Scale US findings Painful side *Suprapinatus tendon*	Grey Scale US findings Pain-free side *Suprapinatus tendon*	Painful side Active movement° (passive movement)	Pain-free side Active movement°
											

1	69	M	L	R	84	**+**	22	Tendinopathy	NAD	F70° (>) Ab85° (>)	F140° Ab130°

2	52	F	L	R	4	**+**	22	Fluid in bursa, tendinopathy	NAD	F54° (>) Ab55° (>)	F120° Ab110°

3	45	F	R	R	1.5	**+**	21	Tendinopathy or PTT	NAD	F100° (>) Ab80° (>)	F160° Ab160°

4	53	F	L	R	132	-	38	Fluid in bursa, ?calcific tendonosis	NAD	F180° (>) Ab160° (>)	F180° Ab170°

5	65	F	R	R	10	**+**	21	Tendinopathy	NAD	F80° (>) Ab120° (>)	F160° Ab160°

6	58	F	L	L	1	**+**	22	Tendinopathy	Tendinopathy	F50° (>) Ab60° (>)	F165° Ab170°

7	56	M	R	R	8	-	45	PTT-articular side	NAD	F110° (>) Ab110° (>)	F170° Ab170°

8	55	F	R	R	12	**+**	22	NAD (biceps tendon changes)	NAD	F120° (>) Ab110° (>)	F170° Ab170°

9	42	F	R	R	12	-	39	Tendinopathy	Tendinopathy	F160° (>) Ab140° (>)	F160° Ab170°

10	57	M	R	R	36	**+**	31	NAD	NAD	F40° (>) Ab30° (>)	F170° Ab170°

11	55	F	R	R	120	**+**	4	FTT and tendinopathy	NAD	F70° (>) Ab40° (>)	F80° Ab50°

12	64	F	L	R	0.75	-	34	Tendinopathy	Tendinopathy	F100° (>) Ab100° (>)	F170° Ab170°

13	45	F	L	L	1	**+**	22	Tendinopathy	NAD	F95° (>) Ab80° (>)	F175° Ab175°

14	56	M	R	R	1.5	**+**	25	FTT and tendinopathy	NAD	F160° (>) Ab90° (>)	F170° Ab170°

15	32	F	L	R	2.5	**+**	31	NAD	NAD	F90° (>) Ab80° (>)	F170° Ab170°

16	33	M	L	R	2	-	23	Tendinopathy or PTT	Tendinopathy	F90° (>) Ab70° (>)	F185° Ab185°

17	32	M	R	R	4	-	25	NAD	NAD	F115° (>) Ab140° (>)	F180° Ab180°

18	38	M	R	R	6	**+**	21	Tendinopathy	Tendinopathy	F80° (>) Ab70° (>)	F180° Ab180°

19	52	M	R	L	8	**+**	16	NAD	NAD	F70° (>) Ab40° (>)	F174° Ab174°

20	45	F	L	R	6	-	28	NAD	NAD	F140° (>) Ab140° (>)	F180° Ab180°

M (male), F (female), L (left), R (right), + (present), - (not present), OSS (Oxford Shoulder Score), NAD (nothing abnormal detected), PTT (partial thickness tear-supraspinatus), ? (possibly), FTT (full thickness tear), F (flexion), Ab (abduction in the plane of the scapula), ° (degrees of movement), > (passive movement greater than active), < (passive movement less than active), = (passive movement equal to active)

Fourteen of the 20 subjects had grey scale ultrasound changes in these tissues in the symptomatic side. Five subjects had grey scale changes in the asymptomatic shoulder. Of the 20 subjects included in the formal analysis, a total of 13 subjects (65%) demonstrated neovascularity in the symptomatic shoulder and 5 subjects (25%) demonstrated neovascularity in the asymptomatic shoulder. The presence and location of any identified neovascularity are detailed in Table 2.

**Table 2:** Power Doppler ultrasound findings and presence, location and grade of neovascularity

Subject	Neovascularity present painful side	Location	Grade	Neovascularity present pain-free side	Location	Grade
1	**-**	-		**-**	-	

2	**+**	peribursal and superficial tendon	I	**-**	-	

3	**+**	peribursal and superficial tendon	II	**-**	-	

4	**+**	superficial tendon	II	**-**	-	

5	**+**	superficial tendon	II	**-**	-	

6	**+**	peribursal and superficial tendon	II	**+**	superficial tendon	II

7	**+**	throughout tendon, mainly superficially	III	**-**	-	

8	**-**	-		**-**	-	

9	**+**	peribursal	I	**+**	peribursal	I

10	**+**	peribursal	I	**-**	-	

11	**+**	peribursal	I	**+**	peribursal	I

12	**+**	superficial tendon	II	**+**	superficial tendon	I

13	**-**	-		**-**	-	

14	**+**	superficial tendon	I	**-**	-	

15	**-**	-		**-**	-	

16	**-**	-		**+**	superficial tendon	I

17	**-**	-		**-**	-	

18	**-**	-		**-**	-	

19	**+**	peribursal	I	**-**	-	

20	**+**	superficial tendon	I	**-**	-	

+ (present), - (not present), Grade I (mild neovascularity with no recognisable vessels), Grade II (identifiable vessels involving less than 50% of the area imaged), Grade III (identifiable prominent vessels involving more than 50% of the area imaged)

On the side with symptoms, of the 13 participants with neovascularity, 6 (46%) demonstrated neovascularity changes only within the tendon, all involving the superior aspect of the supraspinatus tendon. Four people (31%) demonstrated evidence of neovascularity only in the bursa and 3 (23%) in both the bursa and superior tendon. Of the twenty subjects with symptoms, 6 were reported to have a structurally normal supraspinatus tendon on grey scale imaging and of these 3 (50%) had signs of neovascularity. Of these three, 2 were located in the bursa and 1 in the superior (bursal) aspect of the tendon. Fifteen subjects without symptoms had no structural abnormality identified in the tendon or bursa. Of the remaining 5 asymptomatic subjects with structural change, 4 (80%) had evidence of neovascularity either around the bursa or in the bursal (superior) aspect of the supraspinatus tendon.

Of the 13 participants with symptoms and neovascularity, 7 participants were identified as having Grade I neovascularity, 5 with Grade II and one with Grade III. In total there were 13 participants who reported having constant pain and of these 8 (62%) presented with signs of neovascularity. Analysis of the data did not suggest an association existed between pain presentation (constant/not constant) and signs of neovascularity (p = 1, Fischer's Exact test). Both the participant who experienced symptoms for the shortest duration (3 weeks) and the one for the longest period (11 years) had signs of neovascularity and analysis of the data did not suggest an association between neovascularity and duration of symptoms (p = .7, Wilcoxon test). Neovascularity was identified both in the participant with the worst Oxford Shoulder Score (4/48) and the best score (45/48). Again, there did not appear to be an association between the degree of disability assessed by the Oxford Shoulder Score and the presence of neovascularity (p = .6, Wilcoxon test).

The data of six subjects were withdrawn as they did not comply with the inclusion criteria. One due to complete cessation of symptoms between the time of being referred to have a shoulder ultrasound scan and the day of the scan, and another 5 subjects due to the development of bilateral symptoms during the same period.

The subject withdrawn due to complete cessation of symptoms was not found to have neovascularity in either shoulder and of the 5 withdrawn due to bilateral symptoms; two subjects were found to have signs of bilateral neovascularity, one subject demonstrated neovascularity in one shoulder and two subjects in neither shoulder. Information on these 6 subjects is presented in Table 3.

**Table 3:** Demographic and clinical information for subjects not included in the main analysis

Subject	Age (years)	Gender	Painful side	Dominant side	Duration of symptoms	Pain at rest	OSS *most * *painful *	Grey Scale US findings Painful side	Neovasc present	Location	Grade	Grey Scale US **findings Painful ** side	Neovasc present		
					(months)		*0 = worse* *48 = best*	*Right* *s.pinatus * *tendon*	Painful side Right			*Left * *s. spinatus * *tendon*	Painful side Left		
21	43	M	**-**	R	120	**-**	33	NAD	**-**			NAD	**-**		

22	53	M	B	R	36	**+**	20	Tendinopathy	**+**	Superficial tendon	**I**	Tendinopathy	**+**	Superficial tendon	**I**

23	68	M	B	R	3	**+**	24	Calcific tendinosis	**-**			Tendinopathy	**+**	Superficial tendon	**I**

24	69	M	B	R	4.5	**-**	18	Tendinopathy	**+**	Superficial tendon	**I**	Calcific tendinosis and tendinop	**+**	Superficial and deep tendon	**II**

25	58	M	B	R	12	**-**	18	Calcific tendinosis and tendinop	**-**			Calcific tendinosis and FTT	**-**		

26	61	M	B	L	12	**+**	24	NAD	**-**			FTT Tendinopathy	**-**		

M (male), F (female), L (left), R (right), + (present), - (not present), OSS (Oxford Shoulder Score), s.spinatus (supraspinatus), neovasc (neovascularity), NAD (nothing abnormal detected), Tendinop (tendinopathy), PTT (partial thickness tear-supraspinatus), FTT (full thickness tear), B (bilateral), Grade I (mild neovascularity with no recognisable vessels), Grade II (identifiable vessels involving less than 50% of the area imaged).

## Discussion

In the current investigation, the prevalence of neovascularity within the rotator cuff in participants with unilateral symptomatic shoulders was found to be 65%. The corresponding prevalence in the asymptomatic shoulders was 25%. This finding suggests that although neovascularity is more prevalent in the symptomatic shoulder of people with a clinical diagnosis of rotator cuff tendinopathy it may also be present in the painfree side. This in agreement with other studies that have also found a higher prevalence of neovascularisation in tendons with symptoms than those without[39,40,63]. There is however disagreement if neovascularity occurs in painfree tendons. Alfredson et al[40] reported a 100% prevalence of neovascularisation in the Achilles tendon from 25 tendons (24 subjects) with a clinical presentation of mid section Achilles tendinopathy and a 0% prevalence in 20 tendons from 14 subjects without symptoms. In contrast, Cook et al[63] reported that of 26 volleyball players with abnormal grey scale ultrasound findings and concomitant neovascularity in the patellar tendons, 15 (58%) had pain and 11 (42%) did not. This finding suggests that neovascularity may be present in painfree tendons. In addition, Cook et al[63] reported the absence of neovascularity in tendons with a normal structural appearance suggesting that it only occurs together with grey scale ultrasound changes. Zanetti et al[39] reported neovascularity in 30 of 55 (55%) painful Achilles tendons and 1 out of 25 (4%) of asymptomatic tendons. The findings reported by both Cook et al[63] and Zanetti et al[39] suggest that although the prevalence of neovascularity is greater in painful tendons it may also occur in painfree tissue. These observations are supported by the findings of the current investigation with a 40% higher prevalence of neovascularity occurring in painful tendons. The reasons for the difference in findings between Cook et al [63], Zanetti et al [39] and the current study that have all identified neovascularity in painfree tendons and those reported by Alfredson et al [40] are uncertain. Possible reasons may be due to methods, age of subjects, difficulty in making a clinical diagnosis of rotator cuff tendinopathy[64-66] and possibly differences between upper and lower limb tendon loading behaviour and pathology in different populations. It is possible that the presence of neovascularity identified in clinically painfree tendons is an indication of subclinical and emerging pathology. This hypothesis would need to be tested in an appropriately conducted longitudinal investigation. It is also possible that neovascularity occurs in an as yet undetermined manner in the cycle of normal and pathological tendon tissue. However the higher prevalence of neovascularity in painful lower limb tendons[39,40,63] and rotator cuff tendons may indicate that this is an ultrasound sign suggestive of a painful tendon. It does not appear to be a finding that may be used in isolation with confidence.

The higher prevalence of neovascularity in painful rotator cuff tendons found in the current study suggests that 65% (approximately 2 in 3) patients clinically diagnosed with rotator cuff tendinopathy may have US signs of neovascularity. This is of potential relevance as pain scores have been shown to be consistently higher in tendons with neovascularity when compared with those without[39,63]. Additionally, the presence of neovascularity may be of relevance as Ohberg and Alfredson[45] hypothesised that the pain in chronic tendinosis may originate from the new blood vessels and the nerves accompanying the vessels. Furthermore, they suggested that sclerosing the neovessels might reduce pain in chronic Achilles tendinosis. In a study of 10 subjects with painful Achilles tendinosis, colour Doppler ultrasound was used to guide an injection of a sclerosing agent into the tendon. Ohberg and Alfredson[45] reported that eight patients were satisfied with the results of treatment. There was significantly reduced pain during activity [reported on a visual analogue scale (VAS)] and no remaining neovascularisation after an average of two injections. Two patients were not satisfied, and the neovascularisation was reported to have remained. At the six month follow up, the same eight patients remained satisfied and could perform Achilles tendon loading activities as desired. Their mean VAS (pain) scores had decreased from 74 before treatment to 8 (p < 0.01), where 100 is maximum and 0 no pain.

More recently, Alfredson and Ohberg[38] conducted a small randomised double blinded trial that compared the effect of a sclerosing injection or control injection (lidocaine and adrenaline) into the area of neovascularity in painful Achilles tendons. The findings suggested that 5 out of 10 subjects receiving a sclerosing injection reported satisfaction with treatment, where as none of the 10 subjects randomised to receive the control injection reported treatment satisfaction. At the 3 month follow-up neovascularisation was absent in all the painfree tendons. This was not the case for the tendons remaining symptomatic. The sclerosing agent used in this study was polidocanol, which has been widely used in the treatment of varicose veins in the legs and oesophagus, telangiectasia (spider veins), haemorrhoids and gastroduodenal lesions[67-69].

The emerging literature suggests that neovascularisation in the patellar and Achilles tendons may be a potential source of symptoms and elimination of this may help to reduce the expression of pain. The finding that neovascularity occurs in clinically symptom free lower and upper limb tendons, suggests that the relationship is not definitive.

## Limitations

In this study as well as in others [39,63], neovascularity was found in asymptomatic tendons. In the study reported by Alfredson et al[40] no evidence of neovascularity was found in asymptomatic subjects. Alfredson et al[40] used a population of asymptomatic people as the control group whereas in the current study the asymptomatic shoulder served as a reference population. It may be that neovascularity when present in one region has a systemic effect on other regions of the same individual and as such a group of age, gender and activity matched asymptomatic subjects may have been more appropriate. Although, the findings from the current investigation were based on a small study population a clear difference in the expression of neovascularity was identified between the symptomatic and asymptomatic shoulders. Due to the anatomical proximity of the subacromial bursa and the superficial tendon a level of uncertainty exists as to the actual location of the neovessels. There is also a degree of uncertainty as to the reliability of the ultrasound reporting. Future research needs to determine the intra and inter tester reliability of both performing and analysing the ultrasound scans. Additional imaging, histological and biochemical studies are necessary to determine the exact locations of the neovessels. The amount of stretch placed on the tendons and the amount of pressure applied by the ultrasound transducer were not controlled for. Both these variables may affect the findings and efforts should be made in future studies to standardise the ultrasound technique to reduce the potential impact that these factors may have on results.

Exercise, in the form of 2 sets of 20 repetitions of shoulder elevation in the plane of the scapula with a 2.3 kg (5 pound) weight in 31 asymptomatic volunteers (mean age 41 years) significantly increased intra and peritendinous vascularity in the supraspinatus tendon[70]. The amount of activity prior to the ultrasound scan in the current study was not controlled for and although all participants waited for approximately 30 to one hour minutes prior to the scan, and no participant participated in exercise during this period, it is possible that carrying bags, coats and jackets prior to the scan influenced the findings. This variable would need to be investigated to determine its influence in future research.

## Conclusion

Although other studies have demonstrated the intertester reliability to detect neovascularity in the Achilles tendon using power Doppler ultrasonography[60] this was not investigated in this study. This is a limitation and questions the accuracy of the reported prevalence of neovascularity reported in the current study. Respecting this limitation, the findings of this study suggest that neovascularity occurs in subjects with a clinical diagnosis of rotator cuff tendinopathy and to a lesser extent in the asymptomatic shoulder of the same individual. As neovascularity may exist in asymptomatic shoulders caution is necessary when reasoning the potential cause and appropriate therapeutic management for an individual. Future research needs to establish; the reliability of the procedure, as well as the relationship and timing of neovascularity and symptoms as well as any correlation between the reduction of neovascularity and pain relief.

## Competing interests

The authors declare that they have no competing interests.

## Authors' contributions

JSL was the chief investigator, conceived the study, conducted the clinical examination and lead on the analysis and write up. SAR performed the blinded analysis of the US scan and contributed to the analysis and write up. JP and CH participated in the design of the study and conducted the US scans. JDP performed part of the statistical analysis. All authors read and approved the final manuscript.

## Pre-publication history

The pre-publication history for this paper can be accessed here:

http://www.biomedcentral.com/1471-2474/10/163/prepub
